# Raman Flow Cytometry and Its Biomedical Applications

**DOI:** 10.3390/bios14040171

**Published:** 2024-03-31

**Authors:** Jiayang Xu, Hongyi Chen, Ce Wang, Yuting Ma, Yizhi Song

**Affiliations:** 1 Zhejiang University-University of Edinburgh Institute, Zhejiang University, Hangzhou 310058, China; jiayang.21@intl.zju.edu.cn; 2 Edinburgh Medical School: Biomedical Sciences, College of Medicine and Veterinary Medicine, The University of Edinburgh, Edinburgh EH8 9YL, UK; 3 Suzhou Institute of Biomedical Engineering and Technology, Chinese Academy of Sciences, Suzhou 215163, China; 4 Division of Life Sciences and Medicine, School of Biomedical Engineering (Suzhou), University of Science and Technology of China, Suzhou 215163, China

**Keywords:** Raman flow cytometry (RFC), label-free, high-throughput, cellular analysis, cell sorting, biomedical application

## Abstract

Raman flow cytometry (RFC) uniquely integrates the “label-free” capability of Raman spectroscopy with the “high-throughput” attribute of traditional flow cytometry (FCM), offering exceptional performance in cell characterization and sorting. Unlike conventional FCM, RFC stands out for its elimination of the dependency on fluorescent labels, thereby reducing interference with the natural state of cells. Furthermore, it significantly enhances the detection information, providing a more comprehensive chemical fingerprint of cells. This review thoroughly discusses the fundamental principles and technological advantages of RFC and elaborates on its various applications in the biomedical field, from identifying and characterizing cancer cells for in vivo cancer detection and surveillance to sorting stem cells, paving the way for cell therapy, and identifying metabolic products of microbial cells, enabling the differentiation of microbial subgroups. Moreover, we delve into the current challenges and future directions regarding the improvement in sensitivity and throughput. This holds significant implications for the field of cell analysis, especially for the advancement of metabolomics.

## 1. Introduction

**Figure 1:**
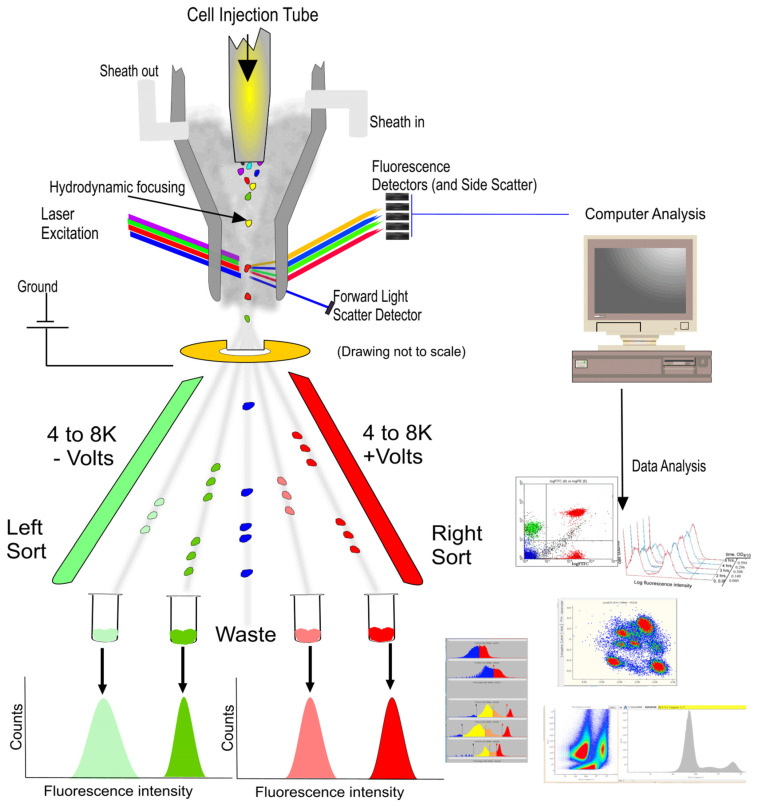
Cell sorting (Left side) and analysis (Right side) of flow cytometry (FCM) [3].

Flow cytometry (FCM) is a cornerstone technology in biomedical research, enabling the high-speed, multiparametric analysis of individual cells’ physicochemical properties [1]. Its development owes much to the pioneering work of Leonard Herzenberg in the late 1960s and early 1970s, who developed the first fluorescence-activated cell sorter (FACS), facilitating the sorting and analysis of cells based on specific fluorescent markers [2]. Today, FCM has evolved into an advanced system that integrates optics, fluid dynamics, and electronic technology, illustrated in Figure 1 [3], which is extensively applied across various fields, including immunology, cancer research, and pathogen detection [4].

During the operation of FCM, cells or particles are first suspended in a suitable medium and can undergo specific molecular tagging through binding with fluorescent antibodies. Subsequently, employing hydrodynamic focusing, the sample stream is directed into a fast and large shear flow, aligning the cells to pass through one by one [5]. Within the laser detection area, cells are irradiated by lasers of specific wavelengths, commonly including 488 nm for exciting green fluorescent protein (GFP) and fluorescein isothiocyanate (FITC), and 633 or 640 nm for red fluorescent protein (RFP) and Cy5 dye [6]. As the laser interacts with the cells, generated scatter light and fluorescent signals are captured by detectors and converted into electrical signals. These signals are then analyzed and processed by computer software, culminating in histograms or dot plots that differentiate between various cell types and states. Forward scatter light reveals the cell size, while side scatter light provides information about the internal structure of cells, and fluorescent signals indicate the presence and quantity of specific molecules inside and outside the cells [3,7,8]. Besides cell analysis and characterization, FCM also facilitates cell sorting. During laser detection, cells that meet predetermined criteria are encapsulated in droplets and assigned different electric charges based on their classification. These charged droplets are then deflected by an electric field and precisely collected into different containers, thereby achieving the goal of selecting and collecting target cell populations from complex cell mixtures [9,10].

However, its reliance on fluorescence labeling methods presents significant limitations. Firstly, certain cell types, such as specific bacteria and microalgae, are unsuitable for fluorescence labeling, and other cells resistant to genetic modification also face challenges with internal protein labeling [11,12]. Secondly, the fluorescence labeling process can alter the cell function and state, which is problematic for studying cellular behavior [13,14]. Meanwhile, labeling might also introduce foreign substances into cells, causing immunogenic reactions, which is a concern in therapies using human-induced pluripotent stem cells (hiPSCs) and chimeric antigen receptor T (CAR-T) cells [15,16]. Thirdly, fluorescence labeling can complicate data analysis, particularly when using multiple labels, as the overlap of emission spectra requires complex compensation techniques [17]. Additionally, non-specific binding and insufficient quantification of fluorescent dyes may further reduce the accuracy of analysis results [18,19]. These drawbacks limit FCM’s ability to characterize cells at the molecular level and its application in clinical cell therapy.

Fortunately, the introduction of Raman spectroscopy into FCM has opened a promising path to overcoming the limitations of traditional FCM [20,21]. By utilizing the inelastic scattering of light, Raman spectroscopy can precisely reveal information about cellular composition. As a “label-free technique”, it avoids altering the natural state of cells or introducing the complexities associated with fluorescent dyes [20,22]. This non-invasive technique has been widely applied in various fields. For instance, using novel non-precious metal Si@SiO_2_ quantum dot Raman probes has revealed a “biochemical fingerprint” of mammalian and cancerous cervical cells, and the use of silver nanoparticle-based active probes in Raman spectroscopy offers an innovative and non-destructive method for identifying drug components [23,24]. As a result, integrating Raman spectroscopy with FCM not only makes cell analysis more accurate and non-destructive, but is particularly beneficial for applications in the field of biomedicine [25]. This review aims to delve into Raman flow cytometry (RFC), an innovative technique combining Raman spectroscopy and FCM. This account begins with an overview of the fundamental principles of Raman spectroscopy, followed by a detailed discussion of the technical specifics, developmental history, and unique advantages of RFC. Subsequently, we explore its significant applications in the biomedical field and discuss the current challenges and potential future directions.

## 2. Basics of Raman Spectroscopy

Raman spectroscopy is an essential technique in molecular characterization, founded on the Raman effect discovered by C.V. Raman in 1928 [26]. This method primarily focuses on the energy transfer during photon–molecule interactions. In this interaction, when molecules encounter monochromatic light from a laser, most photons undergo elastic scattering (known as Rayleigh scattering) and retain their energy [27,28,29]. However, a minor fraction of photons experiences inelastic scattering (the Raman effect), leading to Stokes scattering, where photons lose energy, and anti-Stokes scattering, where photons gain energy [30,31]. The sensitivity of Raman spectroscopy to molecular vibrational modes such as stretching and bending is crucial. These modes are unique to each molecule and determine the Raman activity based on their ability to alter the molecule’s polarizability [31,32]. Raman spectra, which depict the relationship between the intensity of scattered light and frequency shift, provide unique molecular “fingerprints” for identification [26,28,29,30]. Table 1 summarizes the biomolecular assignments of different Raman shifts [33,34]. This specificity, combined with its high sensitivity and non-destructive nature, makes Raman spectroscopy invaluable in fields like chemical analysis, materials science, and forensics [31,35,36,37].

The Raman spectrometer is a precision analytical instrument based on the Raman effect, designed to provide detailed information about molecular structures by measuring the vibrational, rotational, and low-frequency modes of samples [27,38]. It consists of a laser source, a sample illumination system, collection optics, a wavelength selector, and a detector [39,40,41,42,43,44,45]. The laser source emits monochromatic light covering wavelengths from ultraviolet to near-infrared, precisely focused on the sample through lenses and mirrors [40,43,45]. The collection optics system gathers scattered light, including Rayleigh and Raman scattering, while the wavelength selector separates these two types of scattering [39,42]. Detectors, such as charge-coupled devices (CCDs) or photomultiplier tubes (PMT), capture the Raman scattered light and convert it into electrical signals, the strength of which reflects the degree of Raman scattering, thereby generating a Raman spectrum [41,44]. Moreover, high-performance detectors can significantly enhance the resolution and sensitivity of spectrometers.

Building on traditional Raman spectroscopy, a variety of advanced techniques have been developed to enhance the sensitivity, resolution, and specificity of the analysis. For example, surface-enhanced Raman spectroscopy (SERS) uses the surfaces of gold or silver nanoparticles to amplify Raman signals, and is suitable for trace analysis such as detecting environmental pollutants, explosives, narcotics, and identifying biomarkers in medical diagnostics [31,46]. Tip-enhanced Raman spectroscopy (TERS) achieves ultra-high spatial resolution by using metal tips close to the surface of the sample, and is particularly suitable for nanoscale imaging in materials science [47]. Resonance Raman spectroscopy (RRS) uses laser wavelengths that match or are close to the molecular absorption bands to enhance signals of specific vibrational modes, mainly applied in the study of biological molecules like proteins and DNA [48]. Additionally, coherent anti-Stokes Raman scattering (CARS) and stimulated Raman scattering (SRS) involve the interaction of two laser beams, playing significant roles in cell and tissue imaging, as well as chemical analysis of biological samples [21,49,50,51].

Leveraging the non-destructive and highly specific molecular characterization of Raman spectroscopy, we recognize its potential to overcome the challenges of traditional FCM. The emergence and development of RFC is an exciting development. This innovative technology integrates the “label-free” characteristic of Raman spectroscopy with the single-cell manipulation and analysis capabilities of FCM, achieving a non-invasive approach for precise chemical composition analysis of cells, as well as effective cell characterization, differentiation, and identification for sorting. Our discussion will further explore the development of this integration, particularly focusing on how it transcends the limitations of conventional FCM.

## 3. Development and Advantages of RFC

### 3.1. Development of RFC

**Figure 2:**
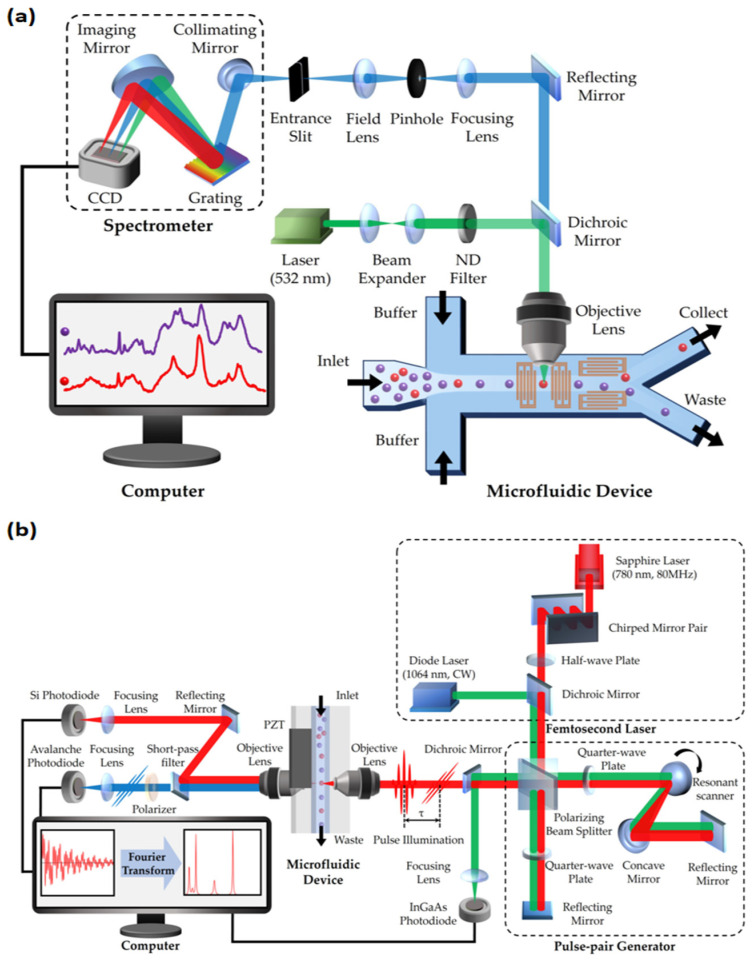
Schematic of two types of Raman flow cytometry (RFC): (**a**) spontaneous Raman flow cytometry; (**b**) Fourier transform CARS (FT-CARS) flow cytometry. CCD: charge-coupled device; ND filter: neutral density filter.

RFC was first reported in 2008 by Dakota A. Watson and his team [20]. This technology overcame the limitations of traditional FCM, especially the challenges posed by the wide emission spectra of fluorescent markers. RFC utilizes the precision and narrow spectral properties of Raman spectroscopy, enhancing the accuracy of multi-parametric characterization of cells and particles. It integrates key elements of Raman spectroscopy and FCM, including the use of helium–neon lasers and CCD detectors. This setup effectively replaces traditional filters and mirrors, enabling the refined detection of Raman scattering spectra. Innovations in RFC also include its optical layout design, featuring square flow cells and high-power lasers, as well as lenses specialized for focusing laser beams and collecting scattered light [20,52]. These technological advancements have given RFC significant advantages in multi-parametric and multi-pathway measurement applications. A typical structure of an RFC system is shown in Figure 2a.

In recent years, the introduction of coherent Raman scattering (CRS) techniques has brought substantial high-throughput benefits to RFC [21,22,53,54,55], as shown in Figure 2b. The weak interaction between light and molecules in previous spontaneous Raman scattering limited the efficiency of processing large groups of cells [56]. Fortunately, the development of various Raman spectroscopy techniques, such as SERS and RRS, has significantly enhanced light–sample interaction. In particular, SRS and CARS are widely introduced in RFC to greatly improve efficiency. These techniques involve complex nonlinear optical processes, significantly enhancing signal strength and spatial resolution while drastically reducing the time required for spectral collection [21]. For instance, the rapid-scanning Fourier transform CARS (FT-CARS) spectrometer developed by Kotaro Hiramatsu’s team achieved a throughput of about 2000 events per second [53], while Yuta Suzuki’s team made significant progress with multi-color SRS microscopy, reaching a throughput of 140 cells per second [55]. This increase in throughput not only accelerates data analysis, but also expands the applications of RFC in high-throughput and real-time analysis, especially in areas such as complex biological process analysis and label-free cancer detection. Table 2 presents a performance comparison of three FCM technologies: Compared to fluorescent FCM, coherent Raman FCM offers non-invasiveness and information richness [21,57], and compared to spontaneous Raman FCM, coherent Raman FCM has higher throughput [20,21]. Despite with significant potential for performance improvement, coherent Raman FCM provides a “high-throughput” and “label-free” method for detecting intracellular molecular levels.

### 3.2. Advantages of RFC

RFC is distinguished by its “label-free” detection capability, which allows for the analysis of cells and molecules without traditional external markers like fluorescent dyes, radioactive labels, or enzymes [9,21]. This method relies on the natural scattering of photons by the sample to generate distinct molecular fingerprints for identification and analysis, offering several benefits outlined below [48].

Firstly, compared to traditional FCM, the “label-free” characteristic of RFC significantly increases the variety of detection signals it can capture, allowing for richer and more specific signals. Traditional FCM is often limited by the availability and types of fluorescent labels. This limitation restricts traditional methods to detecting only a limited range of protein types with limited information [3]. In contrast, RFC can detect a wide array of biomolecules, such as proteins, lipids, and nucleic acids, without labeling [21,58]. This ability to analyze Raman scattering spectra allows for a comprehensive understanding of the cell’s chemical composition, facilitating the collection and analysis of multiple parameters and datasets for deeper insights into cellular states [55].

The second advantage of the “label-free” characteristic is its ability to expand the range of cell types that can be analyzed. For example, it allows for the analysis of challenging environments, like hypoxic tumor microenvironments where cancer cells exhibit limited responses to fluorescent labeling due to poor marker penetration and pH changes affecting marker binding [58]. This method also addresses the difficulties faced with rapidly dividing cells, such as some cancer cell lines, where fluorescent markers may dilute or distribute unevenly during division, leading to inaccurate analyses. Additionally, microorganisms with complex structures, like Staphylococcus aureus and Streptococcus pneumoniae, challenge fluorescent labeling due to their thick cell walls and biochemical compositions that hinder marker penetration [59,60]. Moreover, traditional methods struggle with small or subcellular structures, like ribosomes and lysosomes, due to inadequate labeling [61]. RFC’s capability to detect molecular vibrations without direct labeling overcomes these challenges, offering a more versatile tool for analyzing various cell types.

The non-destructive and non-invasive nature of “label-free” technology constitutes its third major advantage. Traditional fluorescent labeling, requiring high light intensity, can damage cellular structures due to light-induced damage to photosensitive biomolecules and cytotoxicity from invasive labeling, leading to altered cell characteristics and potentially misleading results [21,62,63]. In stem cell research and therapy, fluorescent markers can influence stem cells’ differentiation pathways and reduce therapy effectiveness while posing safety risks by interfering with cells’ natural functions [15,16]. Additionally, in drug discovery and disease modeling using primary cells, fluorescent markers can create artifacts that distort the cells’ true responses to drugs or diseases [64]. In contrast, RFC does not require the introduction of external fluorescent markers and can utilize long wavelengths that cause minimal photodamage to excite the Raman effect, more closely approximating the cell’s authentic state. This is particularly crucial in situations where maintaining cell integrity and function is essential.

Finally, another advantage of “label-free” RFC over traditional fluorescence-based methods is its resistance to photobleaching. Fluorescent labels in conventional FCM degrade in brightness with repeated exposure, compromising long-term study results [65]. However, RFC, which utilizes light scattering interactions with molecules, does not affect molecular energy or structure, circumventing photobleaching issues. This feature makes it especially valuable for prolonged cell culture monitoring, ensuring consistent detection and stability over time.

## 4. Biomedical Applications of RFC

**Figure 3:**
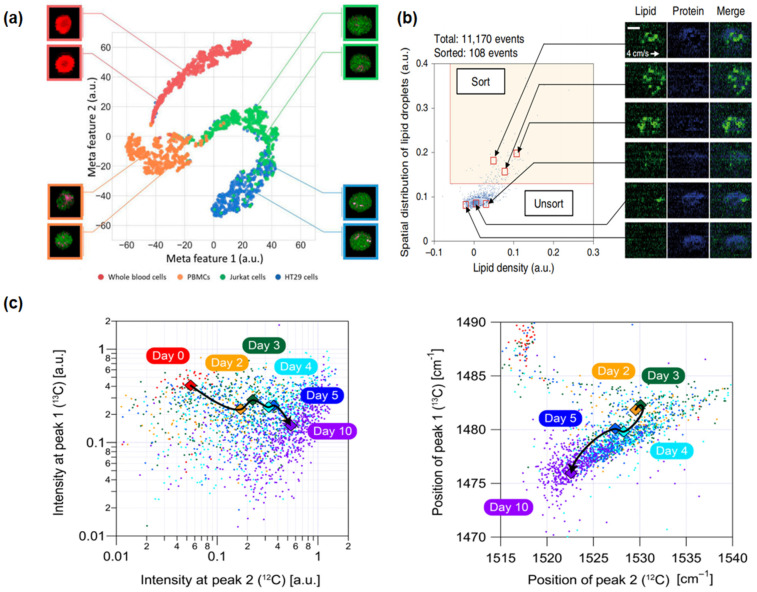
Applications of Raman flow cytometry: (**a**) high-speed multicolor stimulated Raman scattering (SRS) microscopy (cancer cells): This technique involves the characterization of proteins and classification of four types of blood cells using deep learning algorithms [55]. (**b**) Raman image-activated cell sorting (RIACS) (stem cells): This method identifies and maps the distribution of lipids and proteins within cells, facilitating the sorting of cells based on these distributions [58]. (**c**) FT-CARSflow cytometry (microbial cells): This approach is used for tracking and analyzing the metabolic status and photokinetics of cocci, leveraging the capabilities of FT-CARS technology [53].

Leveraging the significant advantages of “label-free” technology, RFC has demonstrated its wide application prospects in the biomedical field, especially in characterizing cells and sorting cells. This section will provide a comprehensive review of the application of this technology in the past fifteen years in research on cancer cells, stem cells, and microbial cells (Section 4.1, Section 4.2 and Section 4.3), focusing on the precise characterization of cell properties and components and cell sorting techniques based on biomolecular characteristics. Some representative results are presented in Figure 3, and important details and parameters of each experiment are listed in Table 3. Furthermore, as key components in addressing the core challenges of disease treatment in the biomedical field, drug development and sensitivity testing (Section 4.4) are also briefly discussed, outlining the progress and potential of RFC technology in these areas, especially in terms of analysis at the cellular level.

### 4.1. Cancer Cells and Cancer Detection

In recent years, RFC has become a crucial tool in cancer research, revolutionizing the way we detect and analyze cancer cells and enhancing our understanding of cancer cell behavior and treatment responses. In 2009, Alexandru S. Biris and his team introduced a pioneering application of in vivo RFC using the unique Raman scattering characteristics of carbon nanotubes (CNTs) [74]. This technique enables real-time monitoring of CNT-tagged cancer cells in live animals and detailed analysis of CNT dynamics in blood, lymph, and tissues, as well as the identification of individual cancer cells in biological environments. It opened new avenues for the non-invasive, real-time tracking of tumor cells. Following this, in 2013, Christina M. MacLaughlin and her team conducted groundbreaking research on the triple detection of leukemia and lymphoma cells using SERS dye-labeled gold nanoparticles, especially in the analysis of malignant B cells [67]. Specific cell surface markers (CD45, CD19, and CD20) were successfully targeted and labeled with polyethylene glycol-coated SERS gold nanoparticles, significantly enhancing the clinical diagnostic capability for cancer cells. Moreover, in 2015, Pallaoro et al. proposed a new method to rapidly identify cancer cells within the RFC framework, combining microfluidics and SERS. This method, based on unique Raman features provided by SERS biotags targeting cell surface markers, with neuropilin-1 as a key biomarker for cancer cells, specifically distinguishes between cancerous and non-cancerous cells in a flowing microfluidic channel. The study demonstrates highly reliable detection of cancer cells at low concentrations, showing the potential for non-invasive monitoring of therapeutic efficacy [68].

In 2019, Yuta Suzuki and colleagues innovated a novel imaging FCM method using a high-speed, multicolor SRS microscope for label-free chemical imaging of cells in flow, as shown in Figure 3a [55]. The system operated at a throughput of up to 140 cells per second, utilizing a flow speed of 2 cm/s and an excitation pump laser wavelength of 790 nm, and precisely classified and characterized cells through deep learning analysis of SRS images. The experiment achieved high-speed, high-precision classification of red blood cells, Jurkat cells, HT29 cells, and peripheral blood mononuclear cells. Recently, in 2023, Xi Xian Wang and his team developed the pDEP-DLD-RFC system, a robust RFC system designed for high-throughput, label-free metabolic phenotype analysis of various cell types, especially suitable for microbial and cancer cells [69]. Utilizing an excitation wavelength of 532 nm for full-spectrum spontaneous single-cell Raman spectroscopy (fs-SCRS), the system effectively described the metabolic states of cells and distinguished different human cancer cell lines based on unique metabolic characteristics, with a throughput of about 30 events per minute for cancer cells. These examples showcase the progress in the application of RFC cytometry for the detection, monitoring, and classification of cancer cells, demonstrating its significant role in future cancer diagnostics and treatment strategies.

### 4.2. Stem Cells and Cellular Therapy

The label-free, high-throughput nature of RFC has revealed its unique advantages in stem cell characterization and sorting. In 2020, a pivotal advancement was demonstrated by Nao Nitta and colleagues with the development of the Raman imaging and cell sorting (RIACS) system [58]. This system, integrating ultrafast multicolor SRS microscopy and advanced real-time image processing technology, achieved perfect cell sorting based on cellular molecular vibration features, particularly in the study of hiPSCs, as shown in Figure 3b. Through RIACS, researchers were able to precisely differentiate hiPSCs in various metabolic states and identify cells grown in different culture mediums, thus maintaining their pluripotency. The RIACS technology demonstrated efficient and precise stem cell sorting capabilities, with a sorting rate of about 100 events per second and a flow speed of 0.04 m/s, relying on pump pulse excitation wavelengths of 790.6 nm and 796.6 nm, and Raman spectral peaks in the high-frequency region (2800–3100 cm^−1^).

Following this, in 2021, R.A. Rocha and his team showcased new progress in using RFC for assessing the biomolecular phenotype and heterogeneity of mesenchymal stem cells (MSCs) [70]. This study, through detailed analysis of intracellular chemical components, revealed biomolecular differences between MSC series, effectively grouping heterogeneous cell populations based on biological functions, such as differentiation capacity, providing important molecular-level information for further research and applications of MSCs. Moreover, in 2011, Flavius C. Pascut and colleagues further expanded the application of this technology by characterizing and sorting cardiomyocytes derived from human embryonic stem cells (hESCs) using a Raman-activated cell sorting method [71]. This technique, depending on high-power lasers capable of sorting about ten cells per second, not only achieved ultra-high specificity and sensitivity, but also opened new possibilities for the enrichment and purification of cardiomyocytes from hESCs. Moreover, coherent RFC has proven to be highly effective in screening cells like hiPSCs and CAR-T cells, which cannot be fluorescently labeled for therapeutic applications [75]. Overall, RFC significantly enhances the precision and efficiency of stem cell research, offering limitless possibilities for future stem cell technologies, such as cell therapy and organ transplantation.

### 4.3. Microbial Cells and Subpopulation Differentiation

RFC has also demonstrated its significant advantages in the field of microbial research. In 2011, Charles H. Camp Jr. and his team were the first to report the use of multiplex coherent anti-Stokes Raman scattering (MCARS) technology for label-free flow cytometric analysis, particularly focusing on the study of brewing yeast, *Saccharomyces cerevisiae* [72]. This technology enabled the rapid and non-invasive detection of lipid content within cells, successfully differentiating yeast cell subpopulations with a throughput of up to 100 spectra per second, showing potential for further increases in processing speed through hardware improvements. Following this, in 2017, the development of SRS-FC by Zhang Chi and colleagues marked another breakthrough in microbial analysis. This technique can differentiate individual *Staphylococcus aureus* bacteria based on their chemical compositions at speeds of up to 11,000 particles per second, demonstrating its exceptional capability for rapid microbial analysis [73].

The work of Kotaro Hiramatsu and his team in 2019 and 2020 further extended the application of RFC in the microbial domain [53,54]. In 2019, they successfully analyzed *Euglena gracilis* using a label-free, high-throughput broadband RFC technique. This method allowed for the rapid characterization of intracellular parameters in more than 1000 cells per second without the need for cell fixation or labeling. By analyzing 10,000 *E. gracilis* under different culture conditions, they found that the distribution of parameters followed a log-normal distribution under a single condition, confirming the technique’s potential to significantly improve the efficiency of biomaterial production. In 2020, they used a rapid-scanning FT-CARS spectrometer to analyze the astaxanthin production and photosynthetic dynamics within *Haematococcus lacustris* cells, as shown in Figure 3c. The study achieved a high throughput of approximately 2000 events per second with a cell flow speed of 20 cm/s, using a titanium–sapphire femtosecond mode-locked laser with a central wavelength of 780 nm as the excitation source. Focusing on broadband molecular vibration spectra, especially the Raman peaks near 1155 and 1520 cm^−1^, allowed for detailed and rapid analysis of the cell components. These studies showcase the tremendous potential of RFC for analysis and classification in the microbial field, which may be extended to related areas, including antibiotic resistance studies, environmental microbiology, food safety, and clinical diagnostics, providing a comprehensive window into the microscopic world [73,74,76].

### 4.4. Drug Discovery and Sensitivity Assessment (Cellular Level)

RFC, due to its ability to directly detect metabolic characteristics and biomolecule variations in cells post-drug treatment, has been leveraged in studying drug toxicity, action mechanisms, and cellular-level efficacy. Ulrich-Christian Schroder and his team developed a RFC system employing dielectrophoresis for bacteria trapping, demonstrating that alterations in specific Raman bands could shed light on vancomycin’s toxicity or antimicrobial impact on *Enterococci* spp. [77,78]. Furthermore, the integration of heavy water (D_2_O) results in the emergence of a distinct Raman C–D band within the 2000–2300 cm^−1^ range [79], the intensity of which serves as an indicator of single-cell metabolic activity. This technique has been corroborated in studies involving cancer cells and bacteria [80,81], marking a progressive shift towards its application in cellular-level drug sensitivity assessments. Previous research primarily focused on analyzing single-cell Raman spectra from either dried bacteria or cells fixed on aluminum-coated slides. Merging Raman microspectroscopy with FCM to detect in situ Raman signals from cells in fluid samples could significantly enhance both the throughput and automation of this process, promising valuable advancements in the field.

## 5. Current Challenges and Future Directions

Although RFC has specific applications in many areas of biomedical science, and its high-throughput, label-free characteristics have made significant contributions to the development of metabolomics, it is important to note that as an emerging fusion technology that has rapidly developed in recent years, it currently faces major challenges and multiple future directions of development. In this part, we will discuss these challenges and prospects for development in detail.

### 5.1. Sensitivity

One of the biggest challenges faced by RFC is sensitivity. Firstly, Raman scattering signals are inherently weak, with the detection limit of Raman spectroscopy typically ranging from molar to millimolar levels [82]. This sensitivity is several orders of magnitude lower than traditional FCM using fluorescence detection. This limitation makes it particularly difficult to detect signals from small or low-concentration samples. Additionally, considering that cells are complex biological samples, they often come with significant noise and signal overlap issues, further obscuring the already weak signals. Although the signal enhancement techniques of SERS and CRS can mitigate this issue to some extent, the current application of RFC technology is still confined to the analysis of concentrated metabolic products and imaging and sorting limited to high wavenumber areas. Meanwhile, the enhancement effect of SERS is unstable and affected by the “memory effect”; CRS faces challenges such as high equipment requirements and the limited penetration depth of coherent light, hindering its further development [55,58]. To overcome this challenge, there is a need for more advanced detectors and optimized laser sources on the equipment side; on the signal processing side, more advanced signal processing and noise reduction algorithms are required in order to improve the signal-to-noise ratio. Additionally, employing multiple analysis strategies, such as multiple Raman active labels, can address the challenges of complex samples. Furthermore, studies have shown that for CRS. sensitivity can be enhanced through methods like heterodyne detection, polarization-selective measurements, and pulse shaping [83,84,85,86,87]. Alternatively, Raman labeling combined with electronic resonance enhancement can increase sensitivity to the micromolar level [88]. These solutions require interdisciplinary collaboration, incorporating research in physical chemistry and other fields, presenting not only a technological challenge, but also a significant direction for advancement.

### 5.2. Throughput

Another major challenge faced by FCM is the issue of speed and throughput. Compared to fluorescence-based FCM, Raman spectroscopy usually requires longer data collection times, significantly limiting the number of cells or particles that can be analyzed per unit time. In recent years, improvements in coherent Raman spectroscopy techniques and instrumentation have significantly increased the throughput of RFC, reaching 100–1000 cells/particles per second, but this is still far below the 100,000 cells/particles per second of traditional FCM [53,73]. Simply increasing the flow rate to improve throughput is not feasible, as it would reduce the detection sensitivity. Therefore, to achieve high-throughput cell analysis, solutions need to be found from the perspectives of instrumentation and imaging technology. Deblurring techniques developed in the field of imaging FCM, such as the virtual freezing method, are expected to increase the effective signal integration time without affecting the flow rate [89]. Improvements in microfluidic focusing can also help enhance the efficiency of FCM measurements. The development of parallel processing techniques and the combination with multi-focus Raman detection to analyze multiple cells or particles simultaneously could also significantly increase throughput [90,91,92]. In terms of high-throughput cell sorting, the application of microfluidic technology, such as reducing the sorting window, is crucial for achieving high throughput without sacrificing sorting purity or yield. Techniques such as surface acoustic waves, dielectrophoresis, and membrane pumps have been reported as fast methods for cell sorting [93,94]. However, it is important to note that some sorting methods may cause damage to cells, so the appropriate sorting technique should be chosen based on the characteristics of the cells and their specific applications.

### 5.3. Instrumental Design and Data Processing

As for instrumental design, Raman flow cytometers are challenged by complexity and high costs, requiring specialized expertise for their operation and maintenance. Key technical challenges include maintaining precise optical alignment and stability in systems integrating lasers, detectors, and flow cells. Balancing laser power is crucial to generate effective Raman signals while preserving delicate biological samples. Future advancements aim at miniaturizing and creating portable devices to expand application scopes. Enhancements in optical systems for better efficiency and stability; modular designs for adaptability; and automated, user-friendly interfaces are anticipated to make the technology more accessible.

Data processing in RFC is also challenged by managing large, complex spectral data, requiring sample storage and sophisticated analysis methods. The difficulty lies in distinguishing overlapping spectral features and reducing background noise, such as fluorescence, for accurate interpretation. Real-time analysis, essential in many applications, is hindered by these computational demands. For instance, an extremely rapid speed finishing spectral pre-processing (including baseline correction and noise reduction) is required for effective microorganism sorting. Future developments focus on integrating AI and machine learning for enhanced speed and accuracy, developing real-time processing with advanced computing resources, and employing cloud computing for scalable data handling. These advancements are vital for maximizing the potential of RFC in areas like rapid diagnostics and detailed cellular analysis.

## 6. Conclusions

In the field of biomedicine, research at the cellular and molecular levels forms the foundation. With the development of next-generation single-cell sequencing technologies, significant advancements have been made in single-cell analysis methods such as genomics, transcriptomics, and proteomics [95]. However, metabolomics research seems to have encountered a developmental bottleneck in recent years. In this field, the destructive nature of mass spectrometry limits its application in longitudinal and subsequent analyses [96]; nuclear magnetic resonance, due to its slow speed, becomes a bottleneck in high-throughput analysis [97]; and fluorescence detection methods may alter the cell state due to the potential toxicity of labels [98].

Fortunately, the advent of RFC has brought unprecedented developmental opportunities to the field of metabolomics. Since the integration of Raman spectroscopy with FCM was first achieved in 2008, this innovative technology has paved a new path for “label-free” detection. Over the past five years, with the continuous advancement of coherent Raman spectroscopy techniques, RFC has made significant progress in the area of “high-throughput” detection. In various fields of biomedical science, it has demonstrated a wide range of applications and outstanding performance. This technology’s ability to precisely characterize and sort cells and molecules has become fundamental in several disciplines, including immunology and regenerative medicine. However, it does face certain challenges, including issues related to sensitivity and throughput, and there is a continuous need for improvements in equipment and optimization of data processing techniques. Looking ahead, the future of RFC is bright and full of potential. As a cutting-edge technology in biomedical innovation, its ongoing development and application will undoubtedly bring profound insights and advancements to medical science, further consolidating its position as a foundational technology in the field of biomedicine.

## Figures and Tables

**Table 1:** Molecular assignment and biomarkers of the Raman bands [33,34].

Raman Shift (cm^−1^)	Vibrational Mode and/or Assignments	Raman Shift (cm^−1^)	Vibrational Mode and/or Assignments
407	Carbohydrates	1124–1128	Cytochrome C
429	Cholesterol ester	1168–1172	Lipids, Tyrosine
490	Glycogen, Chitin	1240	Phosphate (asymmetric)
538	Cholesterol ester	1254–1270	Lipids, Protein, Adenine, Cytosine, Guanine
573	Cytosine, Guanine, Tryptophan	1313–1315	Collagen, Lipid, Guanine
595	Phosphatidylserine	1330	DNA, Phospholipids, Purine
621	Phenylalanine	1361	Guanine
663–671	Guanine, Thymine in nucleotide, Tyrosine	1386–1389	CH_3_ band
726	Adenine, Peptidoglycan	1421	Peptidoglycan
750	Cytochrome C	1480–1491	Guanine, Adenine
781	Cytosine, Uracil in nucleotide	1573–1582	Amide II, Nucleic acid, Peptidoglycan
845–860	Proline, Tyrosine	1583	Cytochrome C
913–915	Glucose, Ribose	1601–1607	Phenylalanine, Tyrosine
1001	Phenylalanine	1616	Tyrosine
1082	Carbohydrates	1655–1676	Protein, Lipid, Unsaturated fatty acids
1102	Phosphate (symmetric)		

**Table 2:** Comparison of traditional (fluorescence) flow cytometry [57], spontaneous Raman flow cytometry [20], and coherent Raman flow cytometry [21]. eps: events per second.

Flow Cytometry Type	Label	Throughput	Information Content	Biological Interference
Fluorescence Flow Cytometry	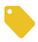 Label Reqiured (fluorescent dye)	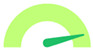 100, 000 eps	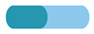 Univariate (up to 12)	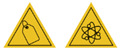 Label Dependent, Photon Dependent
Spontaneous Raman Flow Cytometry	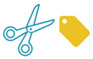 Label Free	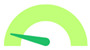 1eps	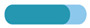 Multivariate (20+)	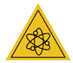 Photon Dependent
Coherent Raman Flow Cytometry	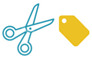 Label Free	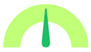 100 eps	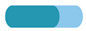 Multivariate (15+)	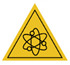 Photon Dependent

**Table 3:** Experimental details and parameter settings for the biomedical applications of Raman flow cytometry.

Sample Type	Biomolecules Detected	Applications	Raman Spectroscopy Type	Excitation Wavelength	Throughput	Reference
Cancer Cells	Carbon nanotubes (CNTs)	Human cervical cancer cells (HeLa) detection	Time-resolved Raman spectroscopy	514 nm, 633 nm, and 785 nm	--	[66]
	Proteins (specific cell surface markers CD19, CD20, and CD45)	Malignant B cells from LY10 lymphoma cell line and primary chronic lymphocytic leukemia cells detection	SERS	638 nm	--	[67]
	Glycoprotein (neuropilin-1, NRP-1)	Cancerous and non-cancerous prostate cells detection and monitoring	SERS	633 nm	--	[68]
	Proteins, lipids, saccharides (paramylon) and pigments (chlorophyll)	Blood samples (cancerous and other cells) detection	SRS microscopy	790 nm and 1030 nm	140 eps	[55]
	Proteins, lipids, and nucleic acids	Human cancer cell lines (bladder (T24), lung (A549), renal (OSRC-2), and breast (MCF-7)) detection and sorting	Spontaneous Raman	532 nm	30 eps	[69]
Stem Cells	Proteins, lipids, and saccharides	High-integrity pluripotent stem cells (hiPSCs) analysis and sorting	SRS microscopy	790.6 nm and 796.6 nm	100 eps	[58]
	Proteins, lipids, and nucleic acids	Bone-derived mesenchymal stem cell (MSC) lines classification	Spontaneous Raman	532 nm	--	[70]
	Proteins (myofibril proteins) and saccharides (glycogen)	Cardiomyocytes derived from human embryonic stem cells (hESCs) analysis and sorting	Spontaneous Raman microscopy	785 nm	10 eps	[71]
Microbe Cells	Lipids	Yeast cell subpopulations classification	Multiplex coherent anti-Stokes Raman scattering (MCARS)	806 nm	100 eps	[72]
	Lipids (triglycerides and lipid-related molecules)	*Staphylococcus aureus* identification and analysis	SRS	1040 nm Stokes beam, 680–1300 nm pump beam	11,000 eps	[73]
	Saccharides (paramylon, a β-1,3-glucan)	*Euglena gracilis* identification and analysis	FT-CARS	780 nm	100 eps	[53]
	Pigments (chlorophyll and astaxanthin)	*Haematococcus lacustris* identification and analysis	FT-CARS	780 nm	2000 eps	[54]

## Data Availability

No new data were created in this work.
